# Fully 3D-Printed Analytical Device Based on a Novel Floating Electrode Mechanism for Sweat Rate Acquisition

**DOI:** 10.1021/acsmeasuresciau.6c00056

**Published:** 2026-05-11

**Authors:** Xing Xuan, Daniel Rojas, Silvia Pérez-Piñero, Vicente Ávila-Gandía, Isabel Maria Diaz Lozano, María Cuartero, Gastón A. Crespo

**Affiliations:** † 16728 UCAM-SENS, Universidad Católica San Antonio de Murcia, UCAM HiTech, Avda. Andres Hernandez Ros 1, 30107 Murcia, Spain; ‡ Faculty of Medicine, Universidad Católica de Murcia (UCAM), Campus de los Jerónimos, Guadalupe, 30107 Murcia, Spain; § Department of Chemistry, 7655 KTH Royal Institute of Technology, Teknikringen 30, SE-114 28 Stockholm, Sweden

**Keywords:** wearables, sweat rate, 3D printing, floating mechanism, one-step manufacturing

## Abstract

We present herein a unique analytical methodology based on a one-step, three-dimensional printed sensing device for measuring sweat rate noninvasively, which allows for the assessment of hydration levels. It comprises a multilayered construction, and diverse designs are investigated, with the printing components consisting of conductive (CB-PLA) and nonconductive polylactic acid (PLA) printable materials. A prominent aspect is the integration of what is termed here as “floating electrodes”, which advantageously modify the overall impedance of the device during sweat flow measurements. To rationalize the mechanism behind this, multiple sensor configurations, including two-, four-, six-, eight-, or multilayer structures, were thoroughly examined. Although multilayer configurations demonstrated the potential to enhance the overall capacity and sensing area, multiplying the number of electrodes in the device effectively resulted in a higher frequency of impedance variations while providing additional sweat rate data points from a single device. In off-body testing, the optimal system (based on four electrodes) exhibits a calibration range of 1–10 μL min^–1^ with a total capacity of 37 μL and a correlation coefficient of *r* = 0.927 between the sensor and timer-based flow validation. With variations oscillating between 0 and 15% as well as a strong correlation (*r* = 0.79–0.93) with the cotton patch gravimetric method, on-body validation utilizing iontophoresis and cycling to generate sweat in the tested subjects demonstrated strong correlation with standard methods. Our findings illustrate the potential of the “floating electrode” concept for scaling up to larger and higher-capacity sweat sensing devices, as it functions effectively in both single- and double-layer designs. In addition, 3D printing technology will allow for on-demand customization of the corresponding analytical devices.

## Graphical abstract

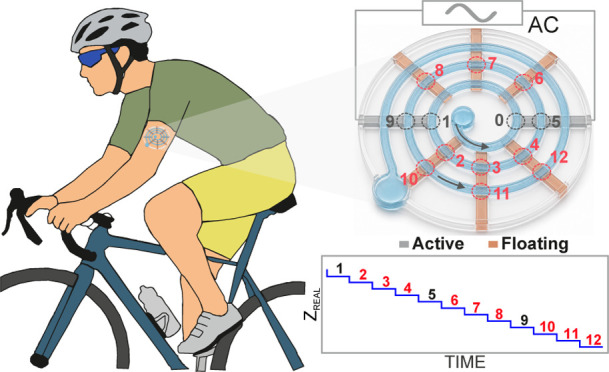

Sweat is a valuable biofluid for noninvasive physiological monitoring, as it may be continuously secreted and collected from the skin surface without disrupting bodily functions during physical exercise.^1–5^ Indeed, sweat offers access to dynamic physiological data, including electrolyte balance, hydration status, and metabolic activity, in contrast to blood or interstitial fluid.^6,7^ Importantly, recent improvements in wearable sensors enable the monitoring of sweat composition, offering new opportunities in sports science, occupational safety, and personalized health assessment.^8–12^ One of the overlooked parameters linked to perspiration is sweat rate (SR), which is defined as the volume of sweat secreted per unit time or per unit skin area.

Advantageously, SR values can be used for different purposes and scenarios, such as (i) assessing thermoregulatory response, thermal strain, dehydration status, and overall body fluid balance;^13^ (ii) estimating total body fluid loss to support individualized hydration strategies and prevent performance decline in athletic activities;^14^ and (iii) normalizing and correcting the measured concentrations of sweat biomarkers (including electrolytes, metabolites, lactate, and glucose) to enable accurate interpretation of sweat analysis data. Beyond these, the practical significance of SR monitoring extends to clinical and industrial areas, where it further supports the early identification of heat stress and electrolyte imbalance during prolonged physical activity or exposure to high temperature environments.^15^

Current strategies for measuring SR include the use of absorbent patches or microfluidic-based sensing techniques. Absorbent patches, such as cotton or filter paper, are simple and cost-effective; however, they accumulate perspiration over prolonged durations instead of offering time-sensitive data. Consequently, these methods are constrained by insufficient temporal precision, being susceptible to evaporation effects and necessitating the use of additional equipment for volume measurement.^9,16,17^ Regarding microfluidic-based systems, they propose a more accurate option, using microchannels to collect and quantify sweat volume dynamically and sometimes incorporating colorimetric or electrochemical detection.^18–24^ Compared with absorbent patch approaches, microfluidic-based devices allow for much better temporal resolution, less evaporation loss, and more accurate output.

Electrical and impedance-based sweat rate sensors have been especially appealing in this category, as they enable continuous monitoring by sensing changes in conductivity, capacitance, or impedance when sweat fills a specific microfluidic area set between electrodes.^25–28^ Direct-current (DC) and alternating-current (AC) measurements have been reported. DC-based approaches are generally simpler in circuitry and signal processing, therefore enabling straightforward implementation.^28^ However, they might result in electrode polarization and electrochemical reactions at the electrode. In contrast, AC or impedance-based methods mitigate polarization effects and provide more comprehensive data regarding ionic transport and dielectric properties.^25^ Nevertheless, these advantages may often come at the expense of higher system complexity and increased cost.

An interesting way to classify SR sensors considers the output style, being either real-time or quasi-continuous. Real-time SR monitoring, where the output reflects instantaneous sweat secretion, has been already demonstrated. For example, the capacitive real-time sensing architectures proposed by Choi et al.^21^ offer instant SR information but often require precise electrode geometries, tight spacing control, or advanced signal conditioning. Notably, most of the reported microfluidic impedance-based SR sensors operate in a quasi-continuous manner, where the sweat rate is calculated from impedance changes as sweat fills the channel. The effective acquisition frequency is determined by the number of electrodes and sensing points. Importantly, quasi-continuous approaches offer improved signal stability and easier calibration compared to real-time devices, but the temporal resolution risks being limited unless additional electrodes (i.e., sensing points) are considered. This can lead to complex designs that are often difficult to manufacture,^25,26^ typically requiring complicated steps like building multiple layers, aligning electrodes precisely, and bonding parts together after printing. Moreover, sensors cost increases, and versatility is rather low; the scale up possibility is also hard to be adopted. A deep inspection of these technologies reveals a persistent manufacturing bottleneck regardless of the transduction mechanism (see Table S1, Supporting Information). The corresponding designs typically require multistep labor-intensive assembly steps, with significant manual fabrication complexity and costs while introducing potential failure points (e.g., delamination), as well as further hindering the scalability required for widespread practical application. Consequently, there is an urgent need to transition toward fully automated manufacturing paradigms that eliminate manual intervention and deal with the mentioned constraints.^29^

Here, we propose a fully integrated, one-step 3D-printed SR sensor based on a novel “floating electrode” mechanism. The conceived architecture utilizes passive electrodes to modulate circuit impedance upon sweat accumulation, effectively decoupling the sensing resolution from wiring complexity. Uniquely, the entire device, including electrodes, insulation, and microfluidics, is fabricated in a single continuous process, eliminating the need for manual postassembly or bonding. Overall, this work establishes a scalable pathway for impedance-based wearable sensing devices. By leveraging the floating electrode concept, the system paves the way for high-frequency data acquisition with minimal structural overhead. The performance of the sensor was validated through on-body trials (stimulating sweat in the subjects by iontophoresis or cycling) to demonstrate its robustness for practical applications in real-time hydration monitoring and personalized health assessment.

## Experimental Section

### Reagents and Instruments

Chloride salts of ammonium (CAS 12125-02-9), magnesium (CAS 7786-30-3), potassium (CAS 7447-40-7), and sodium (CAS 7647-14-5), as well as sodium carbonate (CAS 497-19-8), sodium bicarbonate (CAS 144-55-8), and sodium phosphate (CAS 7558-79-4), were acquired from Sigma-Aldrich. All solutions for the characterization of the SR sensor were produced in 18.2 MΩ·cm doubly deionized water (Milli-Q). Artificial sweat with 60 mM NaCl, 6 mM KCl, 5 mM NH_4_Cl, 0.08 mM MgCl_2_, 2.6 mM NaHCO_3_, and 0.04 mM Na_2_HPO_4_ was used for all characteristic tests. Macroduct sweat collectors (ELITechGroup, Netherlands) and a syringe pump (TYD01-01, Baoding LFT Co., China) were used for testing the sensor performance and the on-body tests. A multimeter to measure the resistance was purchased from RS Components (RS Code 123-1938, Spain). Impedance measurements were conducted at a high frequency of 45 kHz using a Sensit BT instrument (PalmSens BV, Netherlands) to ensure that the system exhibited purely resistive behavior by minimizing capacitive effects.

### Procedure to Fabricate the SR Sensor

The SR sensor was fabricated through a single-step multimaterial fused-filament fabrication (FFF) 3D printing process. The conductive and nonconductive parts were printed using carbon black–polylactic acid (CB-PLA, ref: CDP11705) and PLA (ref: BASF-UF-PLA-TOUGH-NATU-175-750) filaments, respectively. The design, including electrode geometry, microfluidic channel dimensions, and mechanical integration layers, was created in Fusion 360 (Student License, Autodesk, USA) and exported as STL files. The printing protocol was prepared with PrusaSlicer software (Prusa Research, Czech Republic) using a two-nozzle Prusa printer. Printing parameters were set to the following: 100% infill, an extrusion multiplier of 1.1, a nozzle temperature of 230 °C, a bed temperature of 60 °C, and a printing speed of 25 mm s^–1^.

The SR sensor consists of (in this order) a nonconductive PLA substrate layer (0.4 mm thick), conductive CB-PLA electrode layer (0.4 mm), microfluidic layer (0.6 mm), and PLA sealing layer (0.4 mm thick), giving a total thickness of 1.8 mm. The microfluidic channel was printed between the electrode and the top PLA cover. It was 0.6 mm thick and 0.8 mm wide. There were insulating layers between the electrodes with the same thickness as that of the electrode layer (0.4 mm).

The two-electrode design consisted of two active electrodes connected to the impedance readout system, while the four-electrode design had two floating electrodes between the two active electrodes. The six- and eight-electrode designs were built by adding more floating electrodes between the two active ones. Importantly, all of the electrodes were prepared with the same thickness (0.4 mm) to understand how the number of electrodes modulated the impedance response.

A second version of the SR sensor based on a double-layer design was also considered. This had two conductive CB-PLA electrode layers stacked on top of each other and separated by a PLA spacer layer that was 0.4 mm thick. The shape and horizontal spacing of the electrodes in each layer were the same as those in the single-layer design.

### Prevention of Air Bubbles during Measurement

To ensure measurement accuracy, air bubble formation must be prevented, as air bubble entrapment creates volume differences between the actual sweat produced and the occupied microfluidic space. In all experiments, air bubbles were avoided by ensuring a proper seal at the device inlet. For off-body tests, a proper seal was maintained between the syringe pump and the inlet. During on-body trials, pressure- and medical-grade adhesives were applied to maintain complete isolation between the skin and the sensor.

### On-Body Tests Based on Sweat Stimulation via Iontophoresis

Localized sweat stimulation was accomplished using a commercial pilocarpine iontophoresis system following manufacturer guidelines (0.5% pilocarpine gel, 1 mA current, current density of 240 μA cm^–2^, 10 min duration). Prior to applying the electrodes, a drop of water was placed on the skin to minimize irritation. After stimulation, the skin was cleaned, and the SR sensor was applied to the cathodic electrode region for on-body measurements.

### On-Body Tests Based on Cycling

On-body studies were performed under controlled environmental conditions. The environmental chamber was set to a temperature of 22 °C and a relative humidity level of 35%, and the cycling intensity was fixed. Notably, a physician supervised all trials to ensure participant safety throughout the testing. Heart rate, Borg scale rating, and body temperature were collected every 5 min during exercise. Local sweat rate was continuously monitored using the wearable SR sensor placed on the arm. Additional validation measurements were performed using the gold standard cotton patch method. Whole-body sweat loss was determined by measuring nude body weight before and after exercise while minimizing moisture retention from clothing.

Regarding the utilized equipment, heart rate was monitored using a dual ANT+/Bluetooth chest strap (Kalenji, France). Body temperature was recorded using forehead (FTN Infrared, Medisana, Spain) and ear (ThermoScan 7, Braun, Germany) thermometers. Exertion level was measured with the Borg scale (range from 0 to 10). The whole-body sweat loss was calculated from the difference in nude body mass before and after testing using a precision balance with a 10 g accuracy (TMZ, Baxtran, Spain). Hydration status before testing was verified using urine specific gravity measurements obtained with a hand-held refractometer (OPTi Digital Hand-held, Xylem Analytics, UK). The ergometer for the on-body test was a Tack Neo 2T Smart trainer (Garmin, Spain).

Regarding the pretest hydration procedure and protocol, nude body weight was recorded before exercise, and then subjects completed a 10 min warm-up before the application of the SR sensor. After that, participants completed a 30 min cycling task at a fixed intensity (60% of VO_2_ max power). To ensure the accuracy of body weight measurements, participants first removed residual sweat before weighing and urine collection. Consequently, the cool-down phase was excluded to prioritize these assessments.

### Ethical Approval

All experimental procedures, including the use of the wearable SR sensor, Macroduct device, physiological measurements, iontophoresis stimulation, and human tests, were approved by the UCAM Ethics Committee under the permits CE062308 (30 June 2023) and CE072304 (21 July 2023).

## Results and Discussion

### Design of the SR Sensor

Figure 1a shows the fully 3D-printed SR sensor based on two, four, six, and eight electrodes together with its application in two typical on-body test setups. The first on-body setup featured a Macroduct iontophoresis system that induces localized sweating with an electrical stimulation under the guidelines of the manual.^30^ The second setup used exercise-induced sweating during cycling, and simultaneous monitoring of basic physiological indicators (heart rate, skin temperature, and subjective exertion) was performed. In any case, the 3D-printed sensor was attached to the skin with medical tape and connected to an electrochemical workstation. During this test, a blue-colored food dye (brilliant blue E133) was used to track the sweat flow in the microfluidic channel via optical tracing in conjunction with the impedance measurements. Continuous impedance measurements were recorded with the developed device.

**Figure 1:**
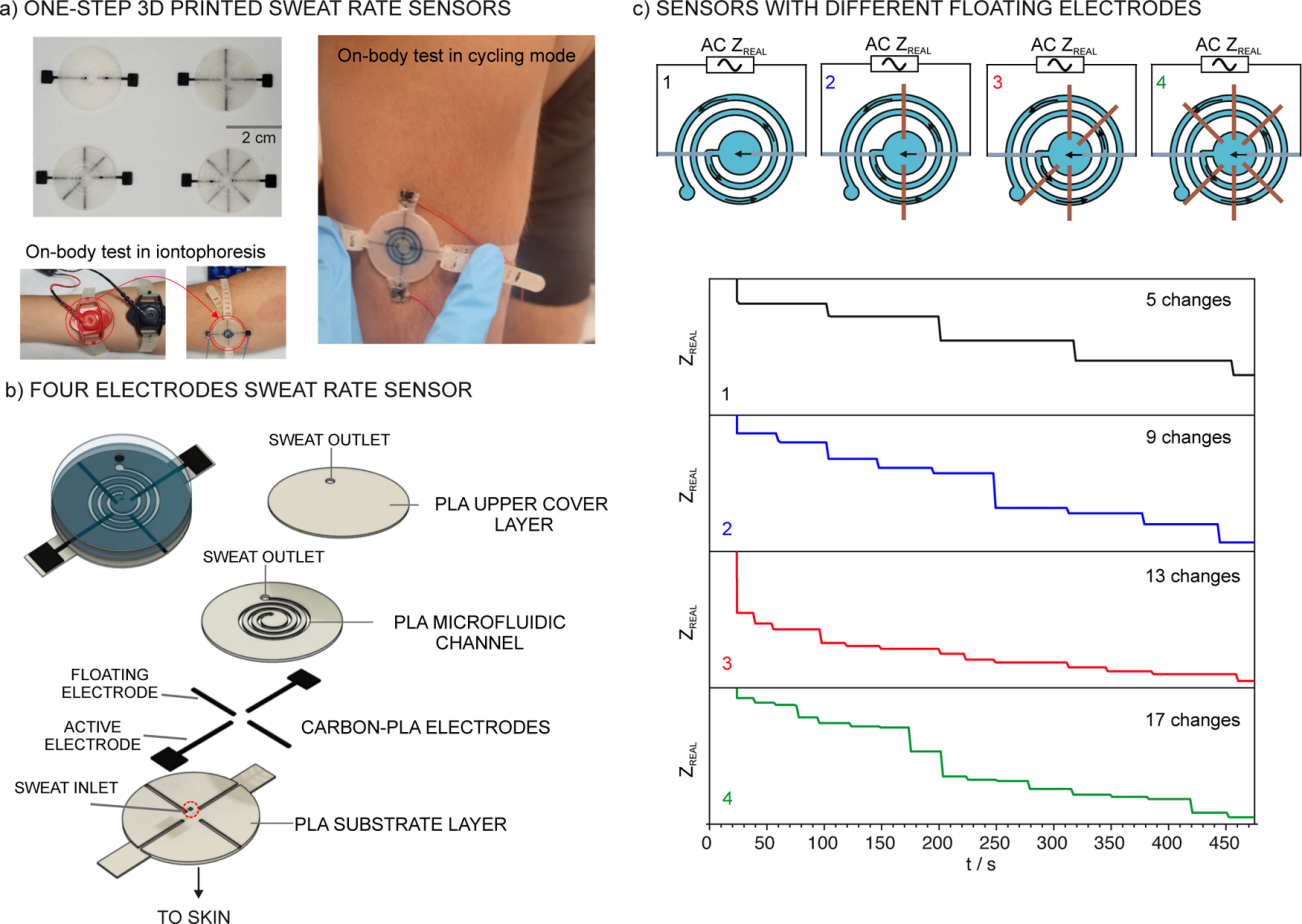
(a) Real pictures of (i) the SR sensor here developed containing two, four, six, or eight electrodes, (ii) iontophoresis-based on-body test, and (iii) cycling-based on-body test. (b) Layer view of the four-electrode SR sensor. (c) Top: Scheme of the SR devices configured with (1) no, (2) two, (3) four, or (4) six floating electrodes. The electrodes with a gray color indicate the active electrodes, which directly connect to the instrument. The electrodes with a brown color indicate the floating electrodes. Bottom: Dynamic impedance outputs observed with the four SR devices at a constant flow rate of 5 μL min^–1^ of an artificial sweat solution.

To obtain the analytical SR device without requiring any manual assembly steps, we developed a unique monolithic manufacturing strategy. Figure 1b depicts the exploded view of the four-electrode SR sensor, which comprises four fused layers: a bottom substrate, a conductive electrode layer, a microfluidic channel for volumetric sampling, and a top sealing layer. Notably, it is here anticipated that the four-electrode configuration is herein established as the optimal one. Unlike conventional microfluidic devices that typically require the bonding of separate components (see Table S1), the entire assembly of the SR sensor was fabricated via a single-step multimaterial 3D printing process. The adopted “click-and-run” approach ensures that both the geometry of the microchannels and the electrode spacing are defined with digital precision during the printing phase, strictly eliminating the variability associated with manual alignment and facilitating further scalable production.

Beyond the technological framework, the core sensing innovation put forward herein is the integration of “floating electrodes” within the microfluidic path (Figure 1c). Using this configuration, only two “active or driving electrodes” (gray) are physically connected to the impedance readout system, with the intermediate electrodes (brown) remaining electrically floating, mechanically present but galvanically isolated from the external circuit. The operational principle exploits the high ionic conductivity of sweat: as the fluid advances through the channel, it sequentially bridges the gaps between the active and the floating electrodes. Upon contact, the floating element becomes ionically coupled to the active circuit, extending the conductive pathway. This mechanism effectively decouples spatial resolution from hardware complexity; increasing the number of floating electrodes proportionally multiplies the number of impedance steps observed. Thus, as the experiment dynamics advances, a certain number of jumps appears as a consequence of the mechanism explained in the next section, with the number of jumps increasing with the number of floating electrodes in the devices, as shown in Figure 1c for SR sensors based on no floating electrodes (configuration 1) as well as two, four, and six floating electrodes (configurations 2, 3, and 4 respectively). Overall, the developed method allows the floating electrodes to be part of the sensing process without needing to make direct electrical contact, which enables the design of the electrodes to be simpler. To better understand how the responses are generated in the four configurations, Figures S1 and S2 illustrate the impedance changes as the sweat sample progressively fills the microfluidic channel at a constant flow rate of 5 μL min^–1^ (experimental time of ca. 450 s). Figure S1 presents the responses of the two- and four-electrode designs, whereas Figure S2 shows the six- and eight-electrode configurations. As observed, for a given time, increasing the number of floating electrodes results in an increasing number of jumps, which will, in turn, result in a higher number of sweat rate values provided for the same dynamic perspiration profile.

### Description of the Mechanism Underlying the Response of the SR Sensor

Figure 2 shows how the 3D-printed four-electrode sweat rate device works in terms of a change in the real impedance output and the equivalent circuit as long as the sweat sample flows through the microfluidic channel and reaches the different electrodes. In essence, the fluid sequentially bridges the gaps between the fixed electrode array, modulating the total system impedance in discrete stages (designated from *R*_01_ to *R*_07_ in the real-time curve). Thus, the impedance profile begins with the initial filling stage (*R*_01_), where the sweat sample bridges the first pair of active electrodes, establishing the baseline ionic conduction pathway (single-resistor configuration). As the flow progresses to the second (*R*_02_) and third (*R*_03_) schemes, the sweat front physically contacts the first and second floating electrodes, respectively. Although these electrodes are not yet fully bridging two points of the circuit, their high conductivity acts as a partial shunt. This introduces a parallel resistance path through the carbon black material, effectively bypassing a segment of the resistive sweat column, as described in eqs 1 and 2.

1 $$R_{02}=R_{01}∥R_{12}$$

2 $$R_{03}=R_{01}∥R_{12}∥R_{23}$$

**Figure 2:**
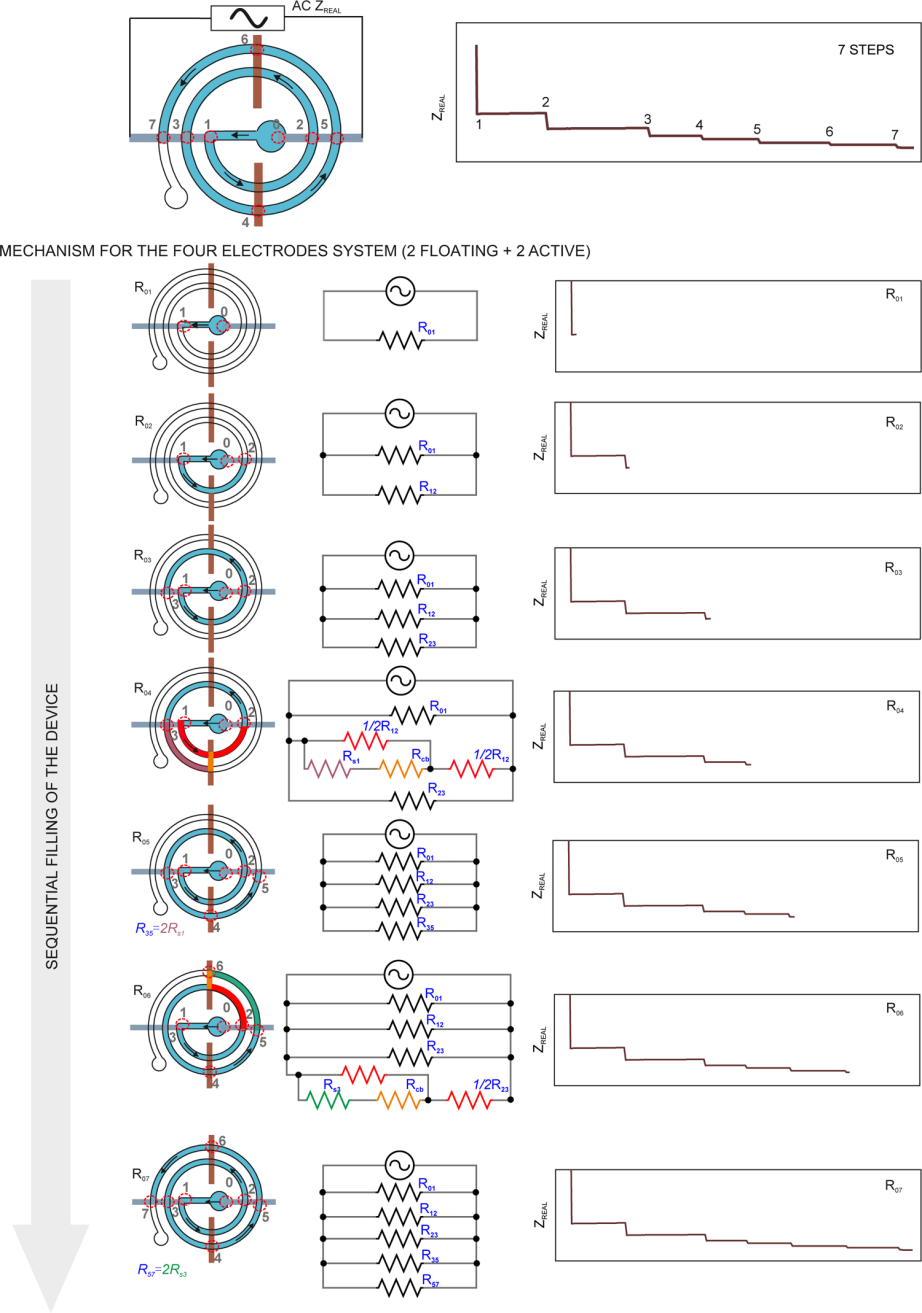
Top: Scheme of the SR sensor based on two active electrodes and four floating electrodes. Areas of contact between the sweat front and the electrodes are numbered. The corresponding dynamic profile for the impedance readout is provided, revealing a total of 7 jumps in the signal for an artificial sweat solution flowing at a constant rate of 5 μL min^–1^. Bottom: Explanation of the sequential filling of the device in terms of electrode contact, equivalent circuit, and changes in the obtained impedance. Explanation for the labels: The two horizontal electrodes (gray color) indicate the active electrodes, which directly connect to the instrument, while the vertical electrodes (brown color) indicate floating electrodes. The numbers and corresponding circles indicate when sweat is in contact with either the floating or active electrode. The light blue color inside the microfluidic channel indicates the sweat sample; the light red, orange, and dark red at the *R*_04_ stage correspond to *R*_12_, *R*_cb_, and *R*_s1_, respectively. The light red, orange, and green at the *R*_06_ stage correspond to *R*_23_, *R*_cb_, and *R*_s3_, respectively.

A critical event occurs at the fourth stage (*R*_04_), where the floating mechanism fully develops. Here, the sweat front bridges the gap to the next segment and inserts a new resistance component, *R*_34_. This stage represents a complex interaction where the floating electrode creates a conductive bridge between all of the previously isolated fluid segments. The equivalent resistance is modeled as a parallel combination involving the carbon black electrode resistance (*R*_cb_), the resistance of the new sweat bridge (*R*_s1_), and the existing ionic path (1/2*R*_12_). As expressed in eqs 3 and 4, this reconfiguration significantly lowers the total impedance, producing the distinct “step” observed in the signal.

$$R_{34}=\left(\frac{1}{2}R_{12}||\left(R_{s1}+R_{\mathrm{cb}}\right)\right)+\frac{1}{2}R_{12}$$ 3

4 $$R_{04}=R_{01}∥R_{12}∥R_{34}$$

Notably, this pattern continues cyclically. At the fifth step (*R*_05_), the fluid reaches the next active electrode, completing a full conduction cycle; the dominant impedance becomes the equivalent resistance between the third and fifth points, modeled as a parallel combination in eqs 5 and 6. Subsequently, the sixth stage (*R*_06_) mimics the behavior of *R*_04_, as a new floating electrode is engaged (eqs 7 and 8), followed by the seventh stage (*R*_07_) producing a similar effect as that noticed at *R*_04_. The resistor *R*_23_ is modified by a new conduction *R*_56_, which can be written as eq 7. Consequently, *R*_06_ can be explained with eq 8. Finally, in the seventh step (*R*_07_), the sweat reaches the second circle of nonfloating electrodes, resulting in the restoration of a conduction path and forming a new resistance *R*_57_ (eq 9). The total impedance at this point is explained by eq 10. Beyond this point, the impedance profile reveals repeating patterns as the sweat continues to propagate through further electrodes; each new cycle may offer a similar sequence of characteristics. To further confirm the hypothesized mechanism for the four-electrode SR sensor, a simplified simulation was performed, as detailed in the next section.

5 $$R_{35}=2R_{s1}$$

6 $$R_{05}=R_{01}∥R_{12}∥R_{23}∥R_{35}$$

$$R_{56}=\left(\frac{1}{2}R_{23}||\left(R_{s3}+R_{\mathrm{cb}}\right)\right)+\frac{1}{2}R_{23}$$ 7

8 $$R_{06}=R_{01}∥R_{12}∥R_{35}∥R_{56}$$

9 $$R_{57}=2R_{s3}$$

10 $$R_{07}=R_{01}∥R_{12}∥R_{23}∥R_{35}∥R_{57}$$

### Impedance Characterization and Frequency Optimization

Figure S3 shows electrochemical impedance spectroscopy (EIS) signals as the device is filled with artificial sweat. This experiment measures the impedance output as artificial sweat occupies multiple channels. Impedance decreases as the number of occupied channels increases due to the mechanism shown in Figure 2. Between 45 and 100 kHz, the impedance exhibits resistive behavior. Figure S4 shows the phase angle at the stage of complete filling with artificial sweat. Between 45 and 100 kHz, the angle is near 0°, which confirms the resistive behavior in this frequency range. The results indicated that capacitive effects, including double-layer capacitance, are negligible, and therefore, 45 kHz is selected for further characterizations of the sensor.

### Numerical Simulation

The developed Python code is provided in the Supporting Information. The ionic conductivity of the sample solution was set to 0.17 S m^–1^, a value within the range reported for human sweat.^31^ Also, this value allows conversion of the microfluidic channel geometry into equivalent ionic resistance values. For simplicity, no variation in the conductivity was assumed. The resistance associated with the printed electrodes, denoted as *R*_cb_, was fixed at 1 kΩ. This value was measured with a multimeter.

Then, Autodesk Fusion software calculated the distances between the electrodes as follows: *L*_12_ = 11.7 mm, *L*_23_ = 15.66 mm, *L*_35_ = 17.95 mm, and *L*_57_ = 21.88 mm. The geometrical properties and the specified sweat conductivity were converted to comparable resistance values to simulate the hypothesized mechanism. *R*_01_ and eqs 1, 2, 4, 6, 8, and 10 were used to find the total impedance at each stage considering the experiment in Figure 2 (top) at a constant flow rate of 5 μL min^–1^. Figure S5 in the Supporting Information overlaps the experimental *R*_0*N*_ values with those calculated by the model.

To quantify the agreement between the experimental measurements and the simulation, the resistance difference at each stage was first calculated by using the experimental values (*R*_exp_) and the corresponding simulated values (*R*_sim_). The results show a small variation compared with the absolute level (around 20–30 kΩ). In the seven stages, the average of the differences is 0.4 kΩ with a standard deviation of 0.3 kΩ. In addition, the experimental values changes were compared with the simulated values changes for all successive stages (*R*_01_–*R*_07_): Figure S6 presents a scatter plot of *R*_sim_ (*y*-axis) versus *R*_exp_ (*x*-axis). The results show a strong linear correlation between the experimental and simulated impedance changes, with a slope of 1.0113 and an intercept of −0.2384. This proximity to the ideal *y = x* relationship indicates that the model successfully captures the dominant physical mechanism.

A slight deviation occurs at stages *R*_04_ and *R*_06_. The difference between the experimental and calculated values are attributed to the following reasons: the floating electrode region is only partially wet; the local channel thickness changes; and there may be delays in ionic equilibrium near the floating electrode, which could lower the effective conductivity at that point. Additionally, small differences between the intended and actual electrode geometries, as well as changes in surface roughness, could affect the actual contact area. Overall, the simulation verified that impedance transitions occur due to the sequential activation of electrodes by the advancing sweat sample and that the model employing parallel resistor networks and contact resistance adequately illustrates the impedance variations during sweat flow. Moreover, the established mechanism agrees with the results obtained with devices based on different numbers of electrodes (Figures S1 and S2). As the number of floating electrodes increases, the amount of measurable impedance changes increases too. Thus, the two-electrode device showed five steps, while the four-, six-, and eight-electrode devices showed nine, 13, and 17 steps, respectively. This pattern confirmed that each additional pair of electrodes created new impedance states that are activated by the flow of sweat into the microfluidic channel.

### Assessment of Different Designs of the SR Sensor

To evaluate how the formulation of the device influences the SR sensor performance, we prepared four different SR sensor designs, as illustrated in Figure 3a. All designs included changes in the structure of the inlet as well as the width of the electrodes. In principle, we expect that these design variations will not change the floating electrode mechanism but may change some signal features. Figure 3b shows that devices with different inlet geometries presented different initial steps, but the total number of steps remained invariable. Also, because of the differences in impedance at the inlet, the total impedance response at each stage (or jump) did change. In essence, *R*_0_ is mainly due to the total volume of sweat sample between the two active electrodes (i.e., a larger volume gives rise to a lower resistance).

**Figure 3:**
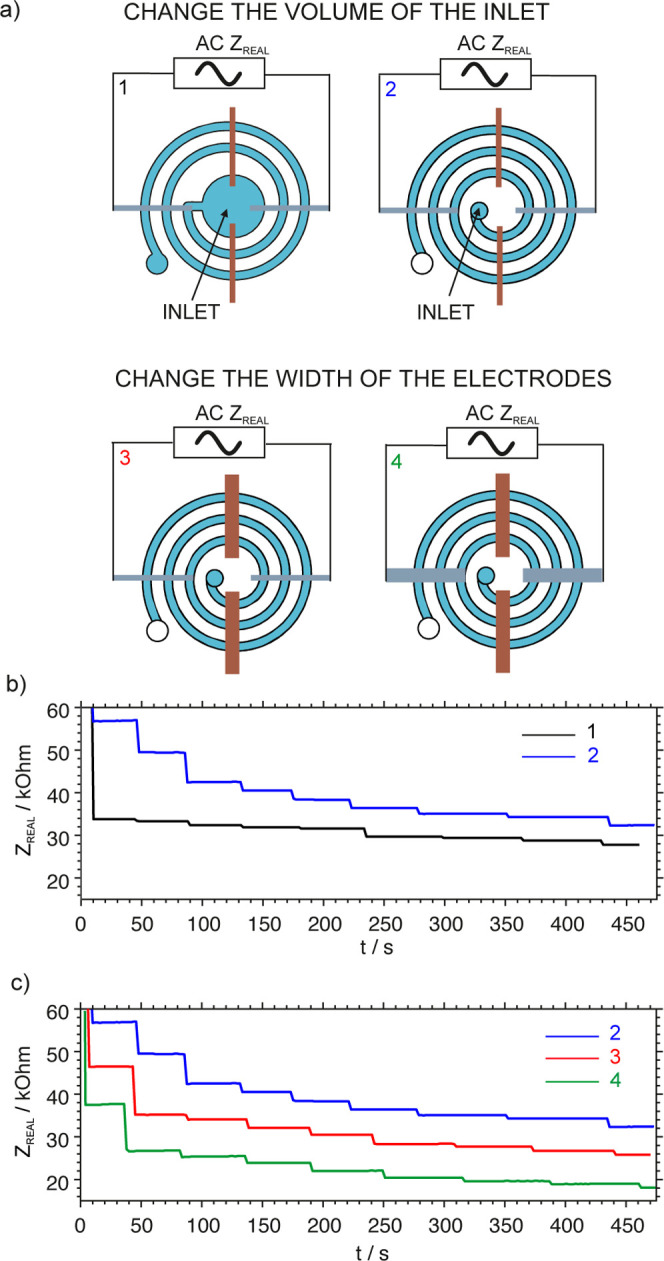
(a) Schemes of the four different designs tested for the SR sensor. Design 1 features a larger inlet chamber (3.0 mm diameter) with an electrode width of 0.4 mm. Design 2 maintains the same electrode width as Design 1 while reducing the inlet diameter to 0.8 mm. Design 3 increases the floating electrode width to 0.8 mm while keeping other parameters consistent. Design 4 increases both the floating and active electrode widths to 0.8 mm. (b) Comparison of the responses observed with designs 1 and 2 with different inlet diameters. (c) Comparison of the responses observed with designs 2, 3, and 4 with different electrode widths. In any case, dynamic impedance outputs were registered at a constant flow rate of 5 μL min^–1^ of an artificial sweat solution.

Figure 3c shows the real impedance changes from different electrode widths (0.4 and 0.8 mm). Wider electrodes made the total impedance lower; however, the pattern of variation remained consistent. Effectively, by making the electrode wider, the conductivity increases, which lowers the impedance (i.e., a wider electrode provides a bigger sample volume between the active electrodes, and hence, a lower resistance is achieved). Overall, the impedance ranges found in the signal provided by the four designs (*n* = 3) revealed some differences influenced by the different initial stage, with the fourth configuration showing the biggest decrease: from 32.1 ± 1.5 to 26.8 ± 0.94 kΩ for design 1, from 57.4 ± 0.6 to 32.6 ± 1.8 kΩ for design 2, from 47.8 ± 1.0 to 26.3 ± 0.5 kΩ for design 3, and from 36.8 ± 0.9 to 18.1 ± 0.5 kΩ for design 4. The results indicate that the floating electrode strategy is effective across all designs, and certain characteristics, such as total resistance and the precise impedance value at each stage, vary according to the conductivity of the electrodes and the initial total impedance.

### Investigation of a Double-Layer Design for the SR Sensor

A potential constraint of the single-layer structure studied until this point for the SR sensor is a limited capacity at high perspiration rates in terms of receiving a high sample volume. For example, considering a test time of 30 min, a collection area of 1.5 cm^2^ in the inlet of the device, and a sweat rate in the range of 1.1–1.6 μL min^–1^ cm^–2^,^32^ the needed internal volume would be ∼50–76 μL versus ca. 40 μL present in the device. Accordingly, a double-layer sweat rate sensor design was also studied, increasing the sample volume to ca. 95 μL, to further understand whether the floating electrode mechanism still works when utilized in a vertical multilayer construction.

Figure 4a,b illustrates the double-layer structure from different perspectives. The design includes two-layered microfluidic channels and electrodes manufactured in a single 3D printing process. The double-layer configuration maintains the same microchannel geometry and electrode width as design 4 in Figure 3. The active electrodes located on the top and bottom layers are electrically connected, while there are four floating electrodes. As shown in the split view in Figure 4a, the sweat sample first enters and fills the top microfluidic layer, operating in the same routine as in the single-layer design. When the sweat sample progresses to the bottom layer and reaches the first bottom electrode, additional impedance drops are generated as new ionic contacts are formed. The output signals can be processed using the same approach used in the single-layer configuration to determine sweat rate values. In contrast to the top layer, the impedance drop in the bottom layer occurs after a longer filling time, followed by shorter intervals, due to the reverse filling direction of the bottom microfluidic channel, as illustrated in Figure 4b.

**Figure 4:**
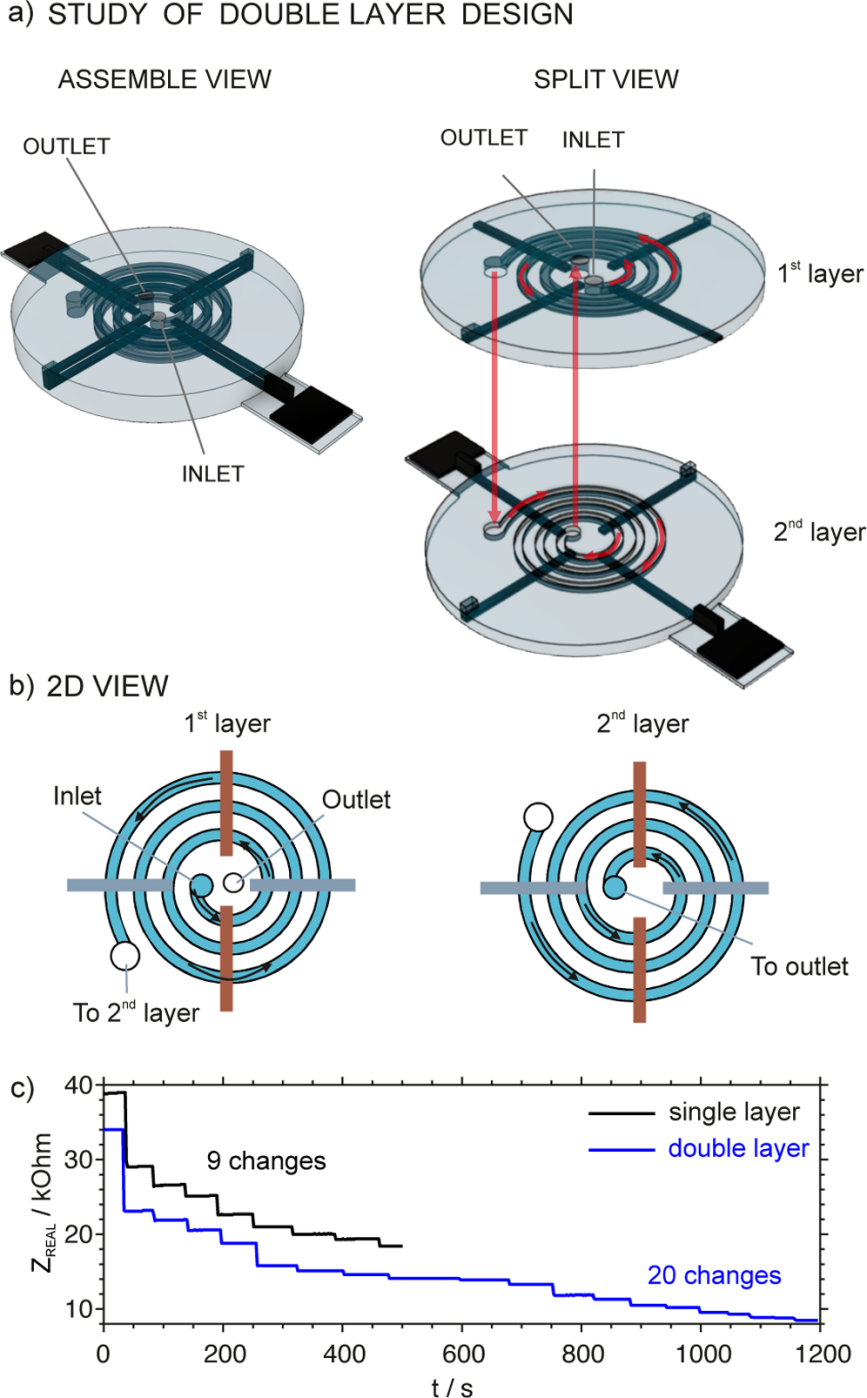
Study of the double-layer design of sweat rate sensors. (a) 3D assemble view (left) and split view (right) of double-layer sweat rate sensors. The red arrows represent the flow inside the microfluidic channel. (b) Schematic drawings of double-layer sweat rate sensors. Black arrows represent the flow inside the microfluidic channel. The gray-colored electrodes represent active electrodes, while the brown-colored ones represent floating electrodes. (c) The comparison of the responses from single- and double-layer designs. The outputs observed with the double-layer design at a constant flow rate of 5 μL min^–1^ of an artificial sweat solution.

Figure 4c shows the responses of the single-layer and double-layer systems at the same flow rate of the sample. The double-layer SR sensor produced a larger total number of impedance drops (20 changes against 9 changes) because the sweat passes through both layers and activates additional floating electrodes (four against two). The magnitude of impedance change at each phase differs from the single-layer design due to a variation in the equivalent circuit system formed by serial and parallel interactions between electrodes. The initial impedance of 22.6 ± 0.4 kΩ was obtained when the sweat sample first built a conductive channel across the active electrodes in the top layer. The smallest impedance of 8.5 ± 0.1 kΩ was obtained when the sweat sample reached the last stage in the bottom layer. These results show that the floating electrode method remains functional in multilayer systems and that more layers enhance the capacity of the sweat rate sensor while maintaining the minimum size during sweat progression. Moreover, this demonstrates that the one-step 3D printing approach can be extended to multilayer designs to improve the sensing capacity without modifying the fabrication methodology. Notably, a balance between the number of floating electrodes, the number of layers, and the gradual decrease of the impedance magnitude as the device is getting filled will mark the final capacity and sensing feature of the device.

### Signal Processing

The signal processing approach to convert raw impedance into sweat rate data followed the general principle employed in previously reported impedance-based SR sensors.^25,28^ Briefly, as shown in Figure S7a for the case of the single-layer four-electrode device, the raw impedance data are dynamically collected while the sweat sample passes through the device at a constant flow rate. Then, the data are converted into the derivative form, and the algorithm identifies the time points and the interval between them considering two successive changes, from *T*_1_ to *T*_8_ (Figure S7b). The time interval corresponding to each impedance change is converted into frequency (*f*) applying the inverse so that *f*_1_–*f*_8_ are generated corresponding to the eight detection zones internally created by the presence of the active and floating electrodes. Ideally, these eight areas should be the same in terms of volume. However, this is not the case because of the consideration of the circular shape of the flow path and the positioning of the electrodes. Accordingly, a calibration of the SR sensor must be created for each path area associated with *T*_1_–*T*_8_ and *f*_1_–*f*_8_.

Because of the outstanding reproducibility in the fabrication of the SR devices, such a calibration graph was built by measuring three identical devices at flow rates ranging from 0.5 to 10 μL min^–1^ and calculating *f*_1_–*f*_8_ *.* Finally, an averaged and universal calibration graph was obtained for each area (Figure S7c shows the calibration curve obtained with the results from the *T*_1_ area). Effectively, analyzing the response under controlled rate changes in each area and creating a fitting of *f* versus the rate, a linear correlation was observed, in which *f*_0,*n*_ is the intercept, *V* is the sweat rate, and *s* is the slope in the corresponding area (*n*):

11 $$f_{n}=sV+f_{0,n}$$

Figure S8 depicts the calibrations averaged for each sensor area at flow rates of 0.5, 1, 3, 5, 7, and 10 μL min^–1^ created with a syringe pump. As the flow rate increased, the channel-filling time was reduced, resulting in shorter time intervals between impedance shifts. As observed, for each step (and area) of the sensor, a different calibration curve was obtained because each impedance change corresponds to a different effective volume in the microchannel: the volume between electrodes increases in later phases, and consequently, each step requires its unique calibration relationship between frequency (or time interval) and flow rate. The following universal calibration lines were obtained, with *R* ^2^ values exceeding 0.99 in all cases:

$$f_{1}\;\left(\mathrm{mHz}\right)=5.7V\;\left(\mu {\mathrm{L min}}^{-1}\right)-0.7$$ 12

13 $$f_{2}\;\left(\mathrm{mHz}\right)=4.3V\;\left(\mu {\mathrm{L min}}^{-1}\right)+0.5$$

14 $$f_{3}\;\left(\mathrm{mHz}\right)=4.1V\;\left(\mu {\mathrm{L min}}^{-1}\right)-0.3$$

15 $$f_{4}\;\left(\mathrm{mHz}\right)=3.9V\;\left(\mu {\mathrm{L min}}^{-1}\right)+0.1$$

16 $$f_{5}\;\left(\mathrm{mHz}\right)=3.6V\;\left(\mu {\mathrm{L min}}^{-1}\right)+0.4$$

17 $$f_{6}\;\left(\mathrm{mHz}\right)=3.4V\;\left(\mu {\mathrm{L min}}^{-1}\right)-0.2$$

18 $$f_{7}\;\left(\mathrm{mHz}\right)=2.9V\;\left(\mu {\mathrm{L min}}^{-1}\right)+0.1$$

19 $$f_{8}\;\left(\mathrm{mHz}\right)=3.0V\;\left(\mu {\mathrm{L min}}^{-1}\right)+0.3$$

Equation[Disp-formula eq12] has a slope of 5.7 ± 0.1 with an intercept of −0.7 ± 0.1. Equation[Disp-formula eq13] has a slope of 4.3 ± 0.1 with an intercept of 0.5 ± 0.2.Equation[Disp-formula eq14] has a slope of 4.1 ± 0.1 with an intercept of −0.3 ± 0.1.Equation[Disp-formula eq15] has a slope of 3.9 ± 0.1 with an intercept of 0.1 ± 0.1.Equation[Disp-formula eq16] has a slope of 3.6 ± 0.1 with an intercept of 0.4 ± 0.1. Equation[Disp-formula eq17] has a slope of 3.4 ± 0.2 with an intercept of −0.2 ± 0.1.Equation[Disp-formula eq18] has a slope of 2.9 ± 0.1 with an intercept of 0.1 ± 0.1, and eq 19 has a slope of 3.0 ± 0.1 with an intercept of 0.3 ± 0.1.

### Temperature and Concentration Influence on Sweat Rate Detection

As mentioned in the mechanism section, the sensor uses a mechanism that converts volume acquisition into discrete impedance transitions. This transduction relies on the frequency of signal peaks rather than the absolute impedance magnitude, which minimizes the impact of sweat conductivity and matrix composition. To evaluate performance under varying conditions, the system was tested across a temperature range of 24 to 40 °C (Figure S9). At a fixed injection rate of 5 μL min^–1^, the maximum variation in time was 3.8 s, resulting in a maximum sweat rate variation of 0.4 μL min^–1^ (Table S2). The influence of chemical composition was also assessed using artificial sweat concentrations ranging from 0.5× to 1.25× (Figure S10). For these ionic strength variations, the maximum recorded time variation was 3.4 s, corresponding to a sweat rate deviation of 0.3 μL min^–1^ (Table S3). These results indicate that while the absolute impedance changes with temperature and concentration, the derived sweat rate remains consistent due to the frequency-based mechanism.

### Off-Body Studies

An off-body validation was performed utilizing the experimental setup indicated in Figure S7. A syringe pump was employed to control the rate of the artificial sweat sample injected in the SR device (single-layer four-electrode device, collection area of 1.5 cm^2^), and a timer was used to record the time gap between the moments when the sweat sample reached each electrode. A blue dye was added to the artificial sweat sample to trace its movement. A total of 54 data points, considering the different *T*_*n*_ of the SR sensor while the syringe pump provided a sweat rate in the range from 1.7 to 7.7 μL min^–1^, were collected and are reported in Table S4. The comparison between the impedance data from the SR device and those obtained with the timer revealed differences ranging from 0.1% to 25.2% with an average of 7.8 ± 5.0%. In addition, the comparison between the SR sensor and the flow rate fixed by the syringe pump showed an average variation of 9.6 ± 5.1%, while the pump versus timer data provided a difference of 8.6 ± 4.2%. In any case, the averaged differences were found to be lower than 10%, and hence, these results validate the performance of the SR sensor.

To further demonstrate the precision of the SR sensor, the repeatability was evaluated by calculating the standard deviation (SD) at fixed injection rates (Table S5). For each flow rate set by the syringe pump, the standard deviations remained remarkably low. At a low flow rate of 1.8 μL min^–1^, the sensor showed a mean of 1.9 ± 0.1 μL min^–1^ (*n* = 8). Similar consistency was observed at 1.3 μL min^–1^ (1.5 ± 0.1 μL min^–1^) and 2.5 μL min^–1^ (2.7 ± 0.1 μL min^–1^). As the flow rate increased, the sensor maintained acceptable precision with recorded mean values of 3.4 ± 0.2 μL min^–1^ (at 3.7 μL min^–1^), 6.0 ± 0.2 μL min^–1^ (at 5.7 μL min^–1^), and 8.6 ± 0.2 μL min^–1^ (at 7.7 μL min^–1^). Finally, at 1.7 μL min^–1^, the sensor provided a mean of 1.8 ± 0.1 μL min^–1^ (*n* = 6). This high level of consistency at fixed flow conditions demonstrates high sensor precision.

### On-Body Studies Based on Sweat Stimulation via Iontophoresis

Iontophoretic sweat stimulation was applied to two healthy male subjects under controlled ambient conditions (22 °C, 40% humidity). Sweat was induced in the arm following the recommendations of the commercial iontophoresis instrument (current: 1.5 mA, current density: 240 μA cm^–2^, pilocarpine concentration: 0.5%). After the stimulation protocol was completed, the SR sensor (single-layer four-electrode device, collection area of 1.5 cm^2^) was placed on the stimulated skin area. Simultaneously, an external timer was set to record the moment when sweat reached each electrode, allowing a comparison between the measured impedance changes and actual sweat arrival times as described for the off-body tests (see Figure 5a).

**Figure 5:**
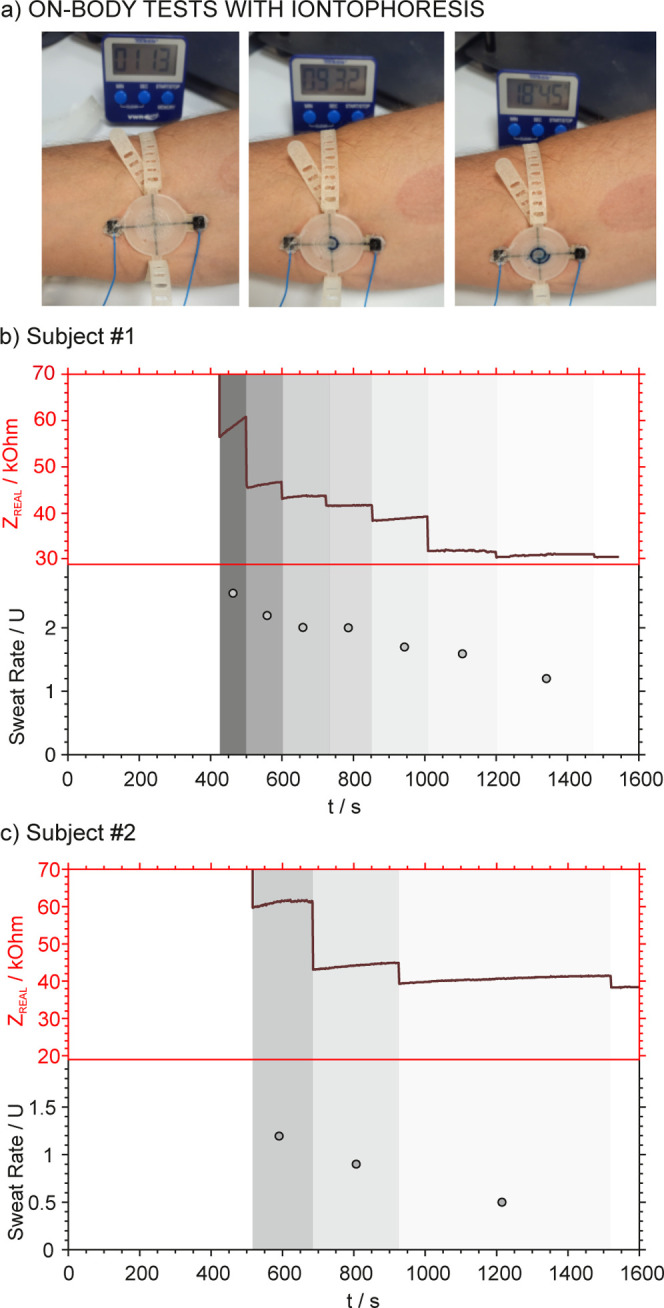
On-body tests linked to iontophoresis for sweat stimulation in the arm. (a) Photos during the on-body test showing the SR device and the timer. (b, c) Results from two subjects. The line and circles represent raw real impedance and the calculated sweat rate from impedance changes, respectively. *U* is expressed in μL min^–1^.

In the first participant (Figure 5b), eight impedance drops (*T*_*n*_) were detected over 25 min after sweat stimulation, resulting in seven SR values. The first ∼500 s of the test contains no signal due to the time required for sweat gland activation following iontophoresis and the subsequent filling of the microfluidic channels. After 25 min, no further impedance changes were detected, corresponding with the end of pilocarpine-induced sweating in the stimulated region. In the second participant (Figure 5c), a slower sweat generation was detected, resulting in four impedance drops and three SR values. Table S6 presents the comparison between the sweat rates provided by the sensor and timer for both subjects, showing an averaged difference of 7.4 ± 4.4% across all 10 data points recorded in the two subjects. The results revealed an acceptable reliability of the sensor in detecting the sweat rate stimulated via iontophoresis.

### On-Body Studies Based on Cycling

In addition to the timer, the gravimetric sweat rate calculation based on sweat collection in a cotton pad was considered. Briefly, a medical-grade cotton pad (5 cm × 5 cm, JFA MEDICA) sealed with hydrofilm tape (10 cm × 10 cm, JIAMU HOME) was weighed before being placed on the skin and used to collect sweat samples during intervals corresponding to two or three impedance drops, establishing synchronization with the sensor data. To prevent evaporation during transportation, the pad was sealed in a Falcon tube following removal from the body for later postweighing.

Figure 6a presents pictures of the SR device upon filling with sweat when placed in the arm of one of the subject. Figure 6b,c depicts the raw impedance data for the two analyzed subjects, the sweat rates produced from the impedance-based calibration curve (*U*_1_ in μL min^–1^), and the sweat rates acquired from the cotton pad (*U*_2_ in mg min^–1^). Notably, according to the perspiration level of the subject, the starting point for the SR measurements varies, and a different number of SR values are found for a given cycling time (i.e., 40 min = 2400 s) and the same training protocol (Figure 6b,c). Because the perspiration level was significantly high in subject 1, a higher number of points was found with respect to subject 2 (eight versus four points corresponding to two consecutive *T*_*n*_), and the SR measurements started quite in advance (ca. 1150 versus 1350 s).

**Figure 6:**
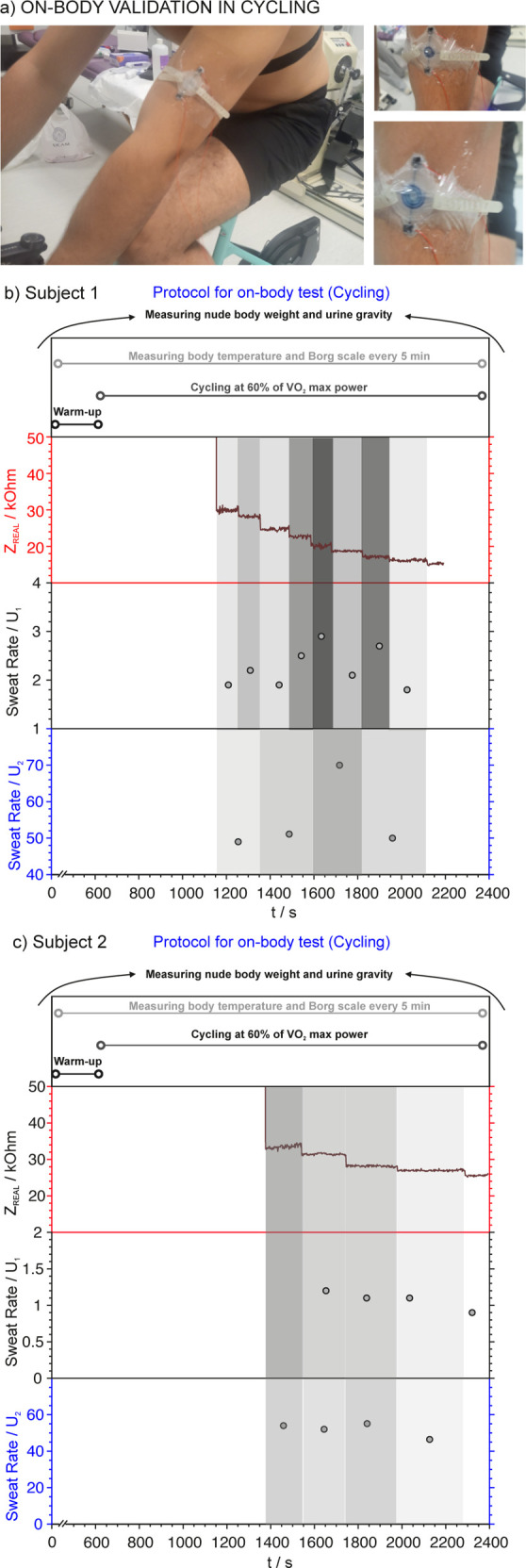
On-body tests linked to cycling-based exercise. (a) Photos of the SR device upon filling with sweat when placed on the arm of one of the subjects. (b, c) Results from two subjects. Each panel (from top to bottom) corresponds to the cycling protocol; raw real impedance data obtained from the SR device; sweat rate calculated from impedance changes using the signal processing method; and sweat rate measured via the cotton patch method. *U* represents units (*U*_1_ = μL min^–1^ and *U*_2_ = mg min^–1^).

For subject 1, the results observed with the three techniques are listed in Tables S7 and S8 in the Supporting Information. Comparing the results from the SR sensor and timer, an average difference of 4.0 ± 1.7% was found, indicating a strong agreement between both methods. Regarding the results from the cotton pad measurements, because the collecting area, body position, and sample procedure rather differ from the SR sensor, a direct comparison of the SR values makes sense only under a correlation study. Accordingly, a linear correlation coefficient of 0.7888 demonstrates a positive relationship between the two methods, considering a typical threshold of 0.8.^33^ Notably, the capacity of the SR sensor was not enough to encompass the perspiration volume during the entire cycling exercise, and hence, it was monitored only until ca. 2200 s.

For subject 2, the perspiration level was slower compared to subject 1, generating a smaller number of drops in the impedance recording, and thus, the acquired SR values were 4 versus 8. The results observed with the three techniques are listed in Tables S7 and S9 in the Supporting Information. Comparing the results from the SR sensor and timer, an average difference of 10.4 ± 7.5% was found, which was slightly higher than 10%. This was caused by the second point, whose value from the SR sensor deviated ca. 20% from that provided by the timer. Then, the coefficient for the correlation between the SR sensor and the pad-based observations was calculated to be 0.927, indicating a strong relationship. Even though the two measurement methods work in different ways, they both show a consistent trend in finding trends and changes in sweat rates.

It is important to note that the on-body results presented here serve as a proof-of-concept. Due to the small number of subjects in this study, these experiments have limitations regarding analytical reproducibility in real-world trials. Future studies will be required to rigorously evaluate these results through repeated on-body measurements on the same subjects under identical, controlled conditions.

To better investigate the physiological context of the two subjects under study and possible uses of the developed SR sensor, Table S10 presents a summary of certain physiological parameters that were measured together with the sweat rate: the total sweat loss, the changes of forehead temperature (f_temp), ear temperature (e_temp), Borg scale, heart rate (HR), and urine density. In contrast to subject 2, who showed a slightly higher total sweat loss (0.66 kg), a smaller temperature change (0.1 °C), a lower Borg scale (6) change, a similar change of heart rate (44 bpm), and a urine density change of 0.0105 SG, subject 1 presented a total sweat loss of 0.62 kg, an increase in forehead temperature of 0.6 °C, a perceived exertion change (Borg) of 9, a change of heart rate (40 bpm), and a urine density change of 0.001 SG. These physiological indicators may offer valuable insights into individual thermoregulatory responses and hydration status. Two key findings can be emphasized from the standpoint of sports performance. First, the differing total sweat loss and perceived exertion of the two subjects may be a sign of different sweating efficiency and temperature tolerance, underscoring the interindividual variability that is essential for customized hydration plans. Second, a novel approach to integrating wearable sweat analysis technology with the real-time evaluation of physical performance and recovery in future research may be offered by the combination of sweat rate profile monitoring with physiological indices like heart rate, body temperature, and urine density. Notably, a larger number of on-body tests are necessary to fully understand the physiology behind the sweat rate results, which is beyond the analytical validation purpose of the present paper.

## Conclusions

We have successfully demonstrated the analytical performance of a 3D-printed monolithic sweat rate device based on a novel floating electrode mechanism. By leveraging multimaterial additive manufacturing, we eliminated the need for manual assembly, bonding, or alignment, steps that traditionally hinder the scalability of wearable microfluidics. The proposed architecture decouples sensing resolution from hardware complexity, allowing for increasing data frequency acquisition through a passive electrode array that modulates the system impedance without requiring additional physical connection. Systematic evaluation of various designs confirmed that this mechanism is robust across different geometric configurations, including high-capacity multilayer structures that double the sensing range without increasing the device footprint. Furthermore, on-body trials during both controlled iontophoresis and dynamic cycling exercise proved the reliability of the sensor in capturing physiological sweat profiles in real time. Overall, the developed click-and-run manufacturing approach addresses a critical bottleneck in the fabrication of epidermal sensors. By providing a low-cost, automated pathway for producing microfluidic sweat rate sensors, this work paves the way for the large-scale integration of sweat rate monitoring into personalized health platforms, athletic performance tracking, and clinical diagnostics.

## Supplementary Material



## Notes

Figures and tables detailing the characterization results of the sweat rate sensor (EIS data, simulated versus experimental data comparisons, sensor responses under varying conditions, repeatability data, and off- and on-body data), comparison of sweat rate sensing technologies between this work and recent literature, and the script used for floating electrode simulations (PDF)
