# Supplementary material for: Fully 3D-Printed Analytical Device Based on a Novel Floating Electrode Mechanism for Sweat Rate Acquisition
Source: ACS Meas Sci Au. 2026 May 11;6(3):825–36. doi: 10.1021/acsmeasuresciau.6c00056 (PMC13281193; doi:10.1021/acsmeasuresciau.6c00056)
Supplement: Supplementary file 1 [file tg6c00056_si_001.pdf]

Supporting information for:

Fully 3D-Printed Analytical Device Based on a Novel Floating Electrodes Mechanism for Sweat Rate Acquisition

Xing Xuan[1], Daniel Rojas[1], Silvia Pérez-Piñero[2], Vicente Ávila-Gandía[2], Isabel Maria Diaz Lozano[1], María Cuartero[1,3,*], Gastón A. Crespo[1,3,*]

[1]UCAM-SENS, Universidad Católica San Antonio de Murcia, UCAM HiTech, Avda. Andres Hernandez Ros 1, 30107, Murcia, Spain.

[2] Faculty of Medicine, Universidad Católica de Murcia (UCAM), Campus de los Jerónimos, Guadalupe, 30107 Murcia, Spain.

[3] Department of Chemistry, KTH Royal Institute of Technology, Teknikringen 30, SE-114 28, Stockholm, Sweden.

*Corresponding authors: mariacb@kth.se; gacp@kth.se

Table of Contents

1. Figures *3*

Figure S1 3

Figure S2 .... Error! Bookmark not defined.

Figure S3 5

Figure S4 5

Figure S5 6

Figure S6 6

Figure S7 7

Figure S8.. 8

Figure S9 8

Figure S10 9

Figure S11 9

2. Tables *10*

Table S1 10

Table S2 10

Table S3 10

Table S4 11

Table S5 12

Table S6 12

Table S7 12

Table S8 12

Table S9 13

Table S10 13

3. Python Code used in the simulation *14*

4. References *15*

1. Figures

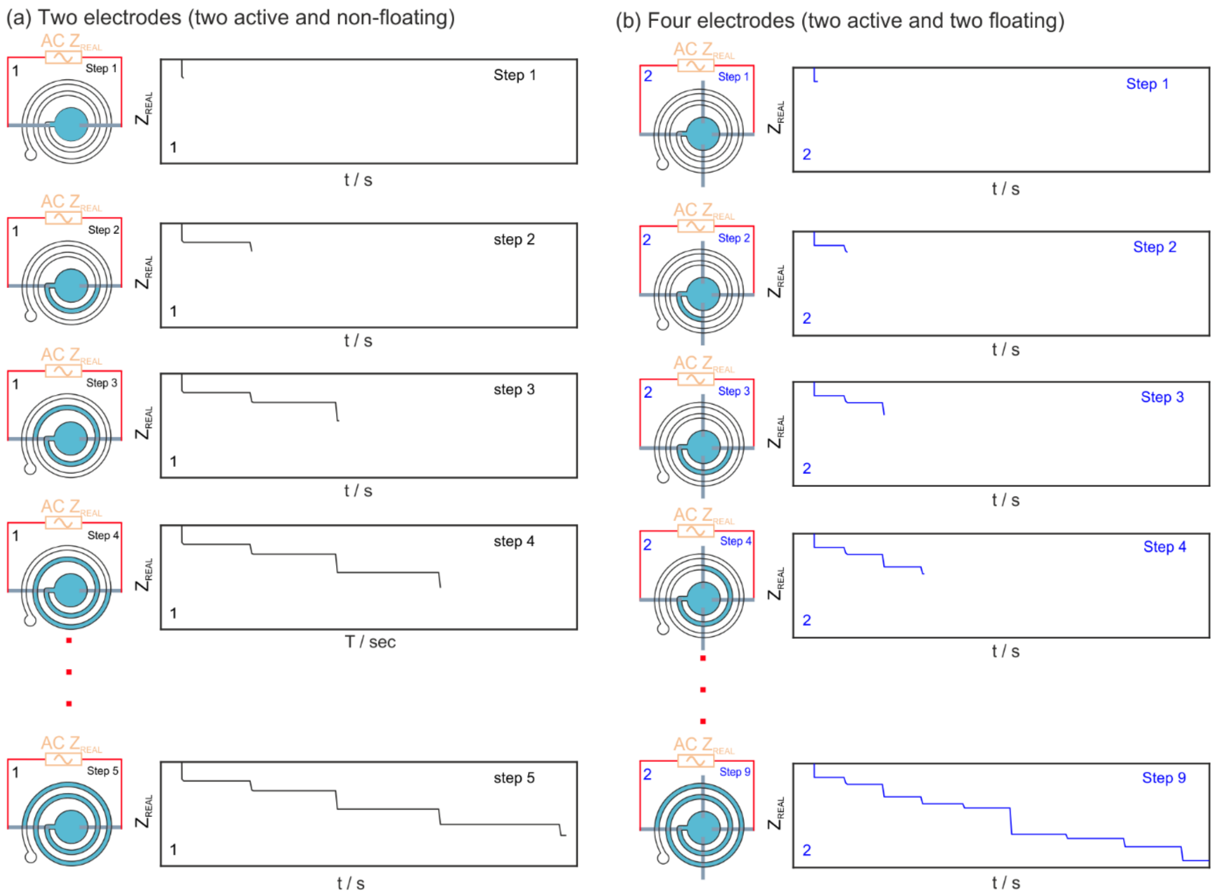

Figure S1. The sensor responses for devices based on (a) two electrodes (two active electrodes and non-floating electrodes) and (b) four electrodes (two active electrodes and two floating electrodes) are shown at each jump step. The blue regions in the device corresponding to each jump (or step) indicate the artificial sweat filling the microfluidic channel.

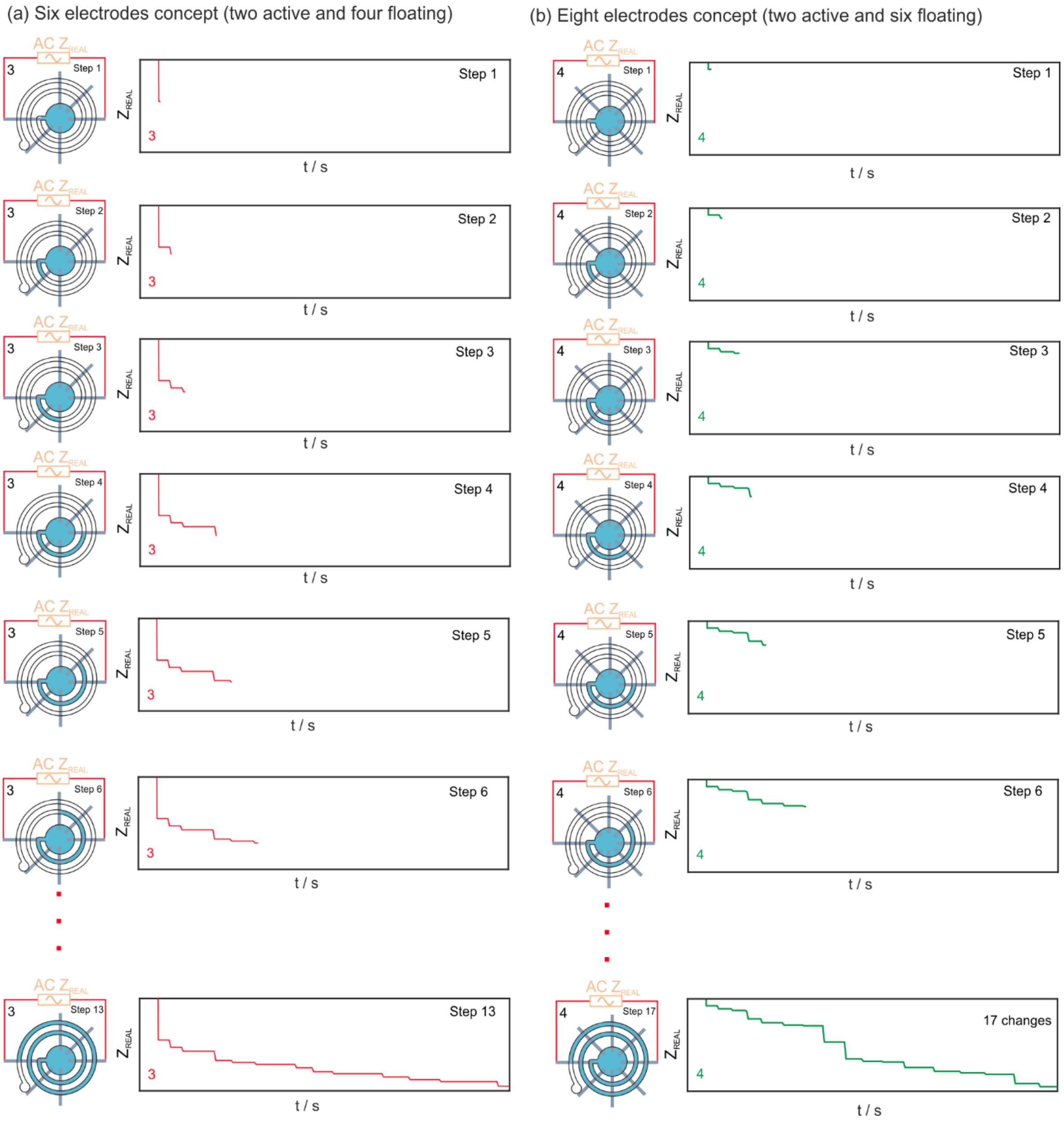

Figure S2. The sensor responses for devices based on (a) six electrodes (two active electrodes and four floating electrodes) and (b) eight electrodes (two active electrodes and six floating electrodes) are shown at each jump step. The six-electrode sensor (left) displays 13 impedance changes, while the four-electrode sensor (right) displays 17. The blue regions in the device corresponding to each jump (or step) indicate the artificial sweat filling the microfluidic channel.

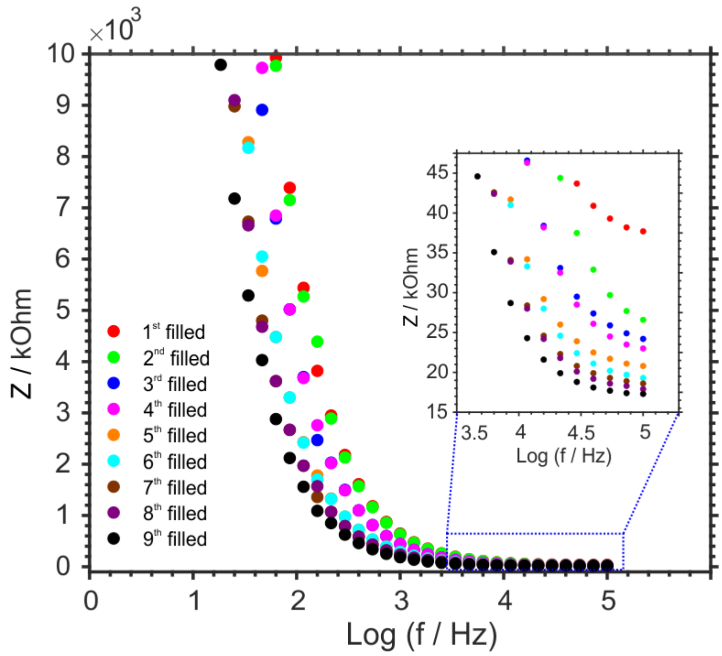

Figure S3. Electrochemical Impedance Spectroscopy (EIS) measurements performed across a frequency range from 100 kHz to 10 Hz at the different stages of the sensor filling with artificial sweat.

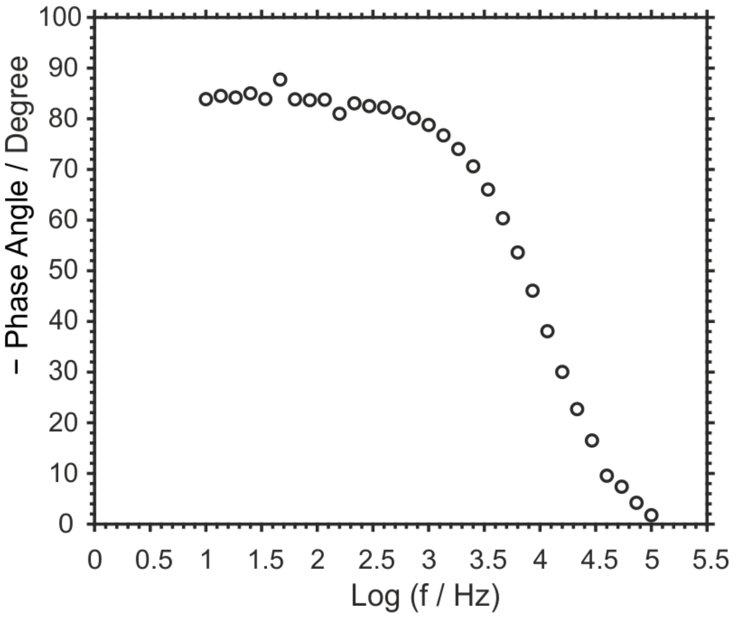

Figure S4. EIS was performed across a frequency range of 100 kHz to 10 Hz at the stage of complete filling with artificial sweat.

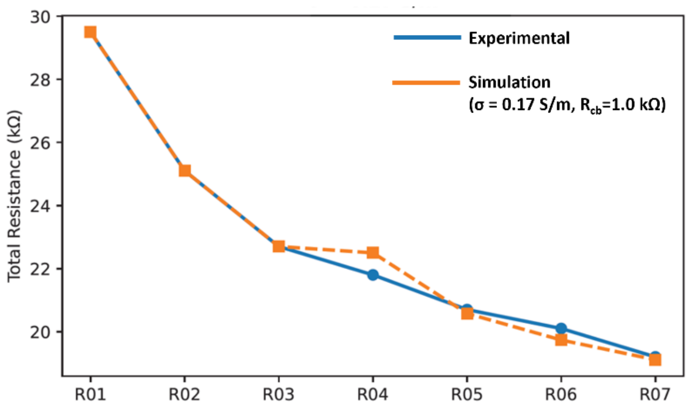

Figure S5. The overlaps of the experimental R_{0N} values with those calculated by the model using the Python script.

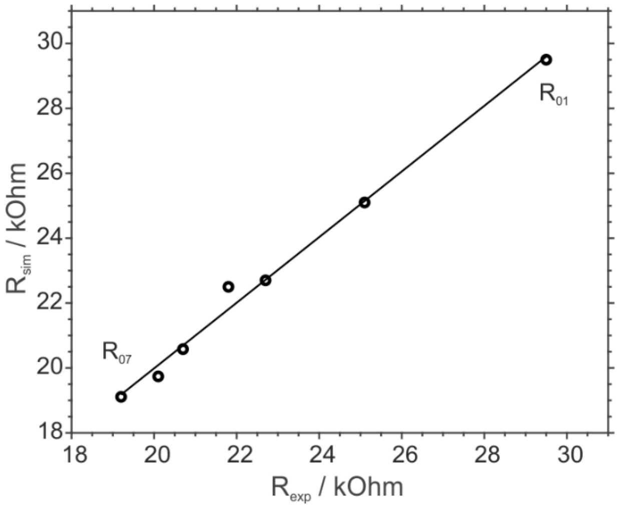

Figure S6. Scatter plot comparing measured experiment values (R_{exp}) from the four-electrode SR sensor and simulated values (R_{sim}). The simulation uses fixed parameters (σ = 0.17 S/m and R_{cb} = 1.0 kOhm).

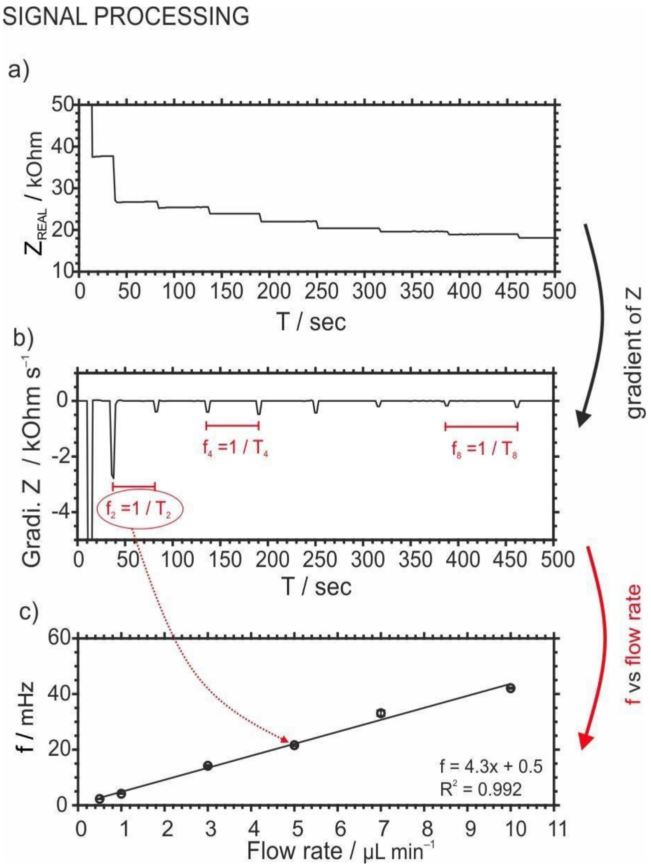

Figure S7. Signal processing to convert raw impedance into sweat-rate values, using the case of the single-layer 4-electrode device. (a) The raw impedance data is dynamically collected while the sweat sample passes through the device at a constant flow rate of 5 µL min^{-1}. (b) The derivative form from the raw impedance data with the interval between them considering two successive changes: from T_1 to T_8 and f_1 *to* f_8. Accordingly, a calibration of the SR sensor must be created for each path area associated to T_1–T_8 and f_1–f_8. (c) An example of the calibration curve obtained with the results from T_1 area.

CALIBRATION OF THE SENSOR WITH FOUR ELECTRODES

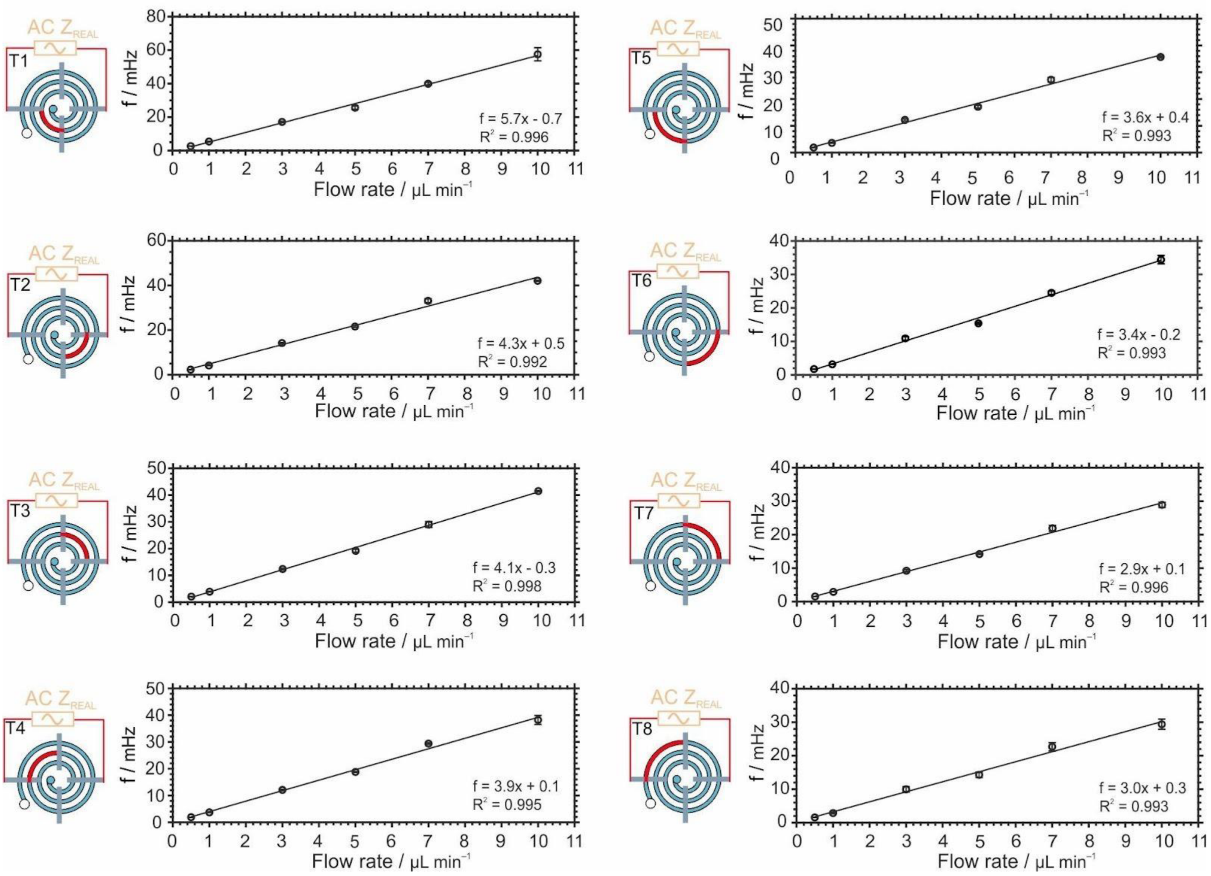

Figure S8. Four-electrode sweat rate sensors were calibrated at flow rates of 0.5, 1, 3, 5, 7, and 10 μL min^{-1} (N = 3 for each rate). Red areas indicate the corresponding effective volumes within the microfluidic channel. A universal calibration curve was generated for each specific area (T_n).

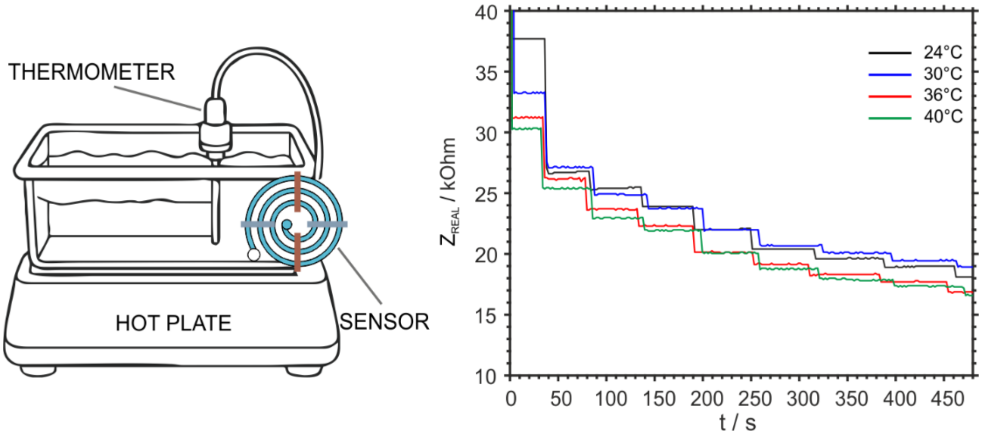

Figure S9. Study of the Influence of different temperature at 5 μL min^{-1} injection rate. The dynamic responses of sweat rate sensor and its experiment set-up. The temperature controlled at the range from 24 to 40 °C.

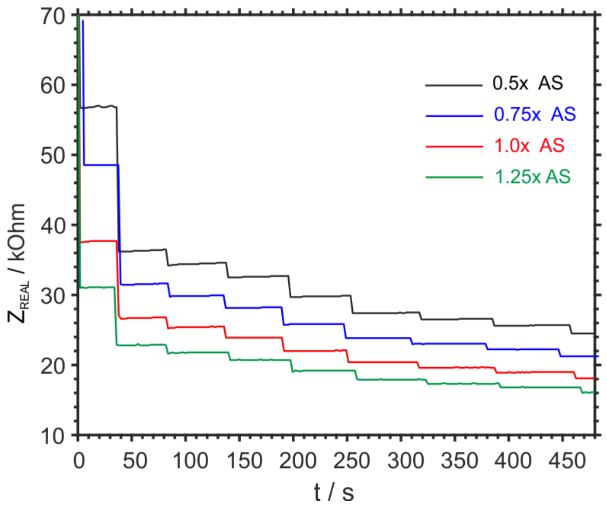

Figure S10. Response of the device to five samples containing different ionic strengths of the artificial sweat: 0.5X, 0.75X, 1.0X, and 1.25X at an injection rate of 5 μL min^{-1}.

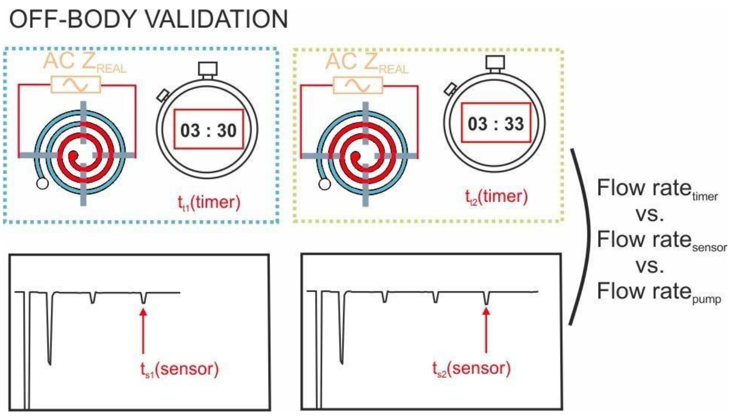

Figure S11. Off-body measurements using the sweat rate sensor were performed using a syringe pump to inject artificial sweat into the single-layer four-electrode device. In parallel, a timer recorded the intervals for the sweat sample reaching each electrode.

2. Tables

Table S1. Comparison of sweat-rate sensing technologies cited in this work.

Operating principle	Manufacturing method	One-step fabrication	Assembly / bonding required	Validation method	Published year	Ref
By capacitance changes	Screen printed; 3D printed	No	Multilayers/ adhesive tape bonding	Macroduct	2025	1
By conductance changes	Laser cut; flexible printed circuit board (FPCB) process	No	Multilayers/ adhesive tape bonding	No	2024	2
By colorimetric	laser and die cutting	No	Multilayers/ glue bonding	Cotton patch	2020	3
By capacitance changes	laser cutting	No	Multilayers/ adhesive tape bonding	Macroduct	2020	4
By humidity changes	3D-printed	No	Multilayers/ No bonding required	No	2023	5
By capacitance changes	Laser cutting	No	Multilayers/ adhesive tape bonding	No	2023	6
By impedance changes	Laser cutting	No	Multilayers/ adhesive tape bonding	No	2023	7
By impedance changes	Screen printed	No	Multilayers/ adhesive tape bonding	No	2023	8
By impedance changes	3D-printed	Yes	Multilayers/ No bonding required	Cotton patch	This work	This work

Table S2. Influence of different temperature at 5 μL min^{-1} injection rate. Considering the different T_n of the sensor at the temperature range from 24 to 40 °C (A total 4 different temperature setting). T_n represents the time interval corresponding to each impedance change.

**T_n**	**Time variations (sec)**	**Sweat rate variations (μL min^{-1})**
1	1.6	0.2
2	3.7	0.4
3	1.0	0.1
4	2.5	0.2
5	1.6	0.1
6	3.8	0.3
7	2.8	0.2
8	2.6	0.2

Table S3. Response influence of the sensor to four test solutions containing different chemical composition of the artificial sweat: 0.5X, 0.75X, 1.0X, and 1.25X at an injection rate of 5 μL min^{-1}. T_n represents the time interval corresponding to each impedance change.

T_n	Time variations (sec)	Sweat rate variations (μL min^{-1})
1	1.9	0.3
2	1.0	0.1
3	2.6	0.2
4	2.3	0.2
5	1.2	0.1
6	1.9	0.1
7	1.9	0.1
8	3.4	0.2

Table S4. The results of off-body tests measuring with the sweat rate sensor and the timer methods A total of 54 data points considering the different T_n of the sensor in the range from 1.7 to 7.7 μL min^{-1}.

Sensor (μL / min)	T_n	Sensor (μL / min)	Timer (μL / min)	Pump (μL / min)	Sensor vs. Timer (%)	Sensor vs. Timer (%)	Photo vs. Timer (%)
2.0	T_1	2.0	1.9	1.8	2.4	8.2	5.9
1.9	T_2	1.9	2.0	1.8	5.1	3.5	8.2
2.0	T_3	2.0	2.0	1.8	0.5	10.8	10.4
1.8	T_4	1.8	1.9	1.8	2.6	2.2	4.7
1.9	T_5	1.9	1.8	1.8	5.5	4.4	1.2
2.0	T_6	2.0	2.1	1.8	4.2	10.1	13.8
2.0	T_7	2.0	1.9	1.8	0.5	8.1	7.6
1.8	T_8	1.8	2.0	1.8	10.2	2.6	11.6
1.6	T_1	1.6	1.5	1.3	1.9	17.0	15.5
1.4	T_2	1.4	1.5	1.3	3.9	8.5	12.0
1.6	T_3	1.6	1.6	1.3	0.4	17.0	16.7
1.4	T_4	1.4	1.4	1.3	0.7	6.4	7.1
1.4	T_5	1.4	1.4	1.3	1.9	7.5	9.2
1.6	T_6	1.6	1.6	1.3	1.5	19.5	18.3
1.5	T_7	1.5	1.5	1.3	1.9	13.2	14.8
1.4	T_8	1.4	1.5	1.3	2.5	9.5	11.7
2.7	T_1	2.7	2.8	2.5	2.5	7.9	10.1
2.7	T_2	2.7	2.7	2.5	1.8	8.5	6.9
2.8	T_3	2.8	2.8	2.5	0.4	10.6	10.2
2.6	T_4	2.6	2.7	2.5	2.8	3.9	6.5
2.7	T_5	2.7	2.7	2.5	0.4	8.6	8.2
2.8	T_6	2.8	2.9	2.5	1.5	12.1	13.4
2.8	T_7	2.8	2.8	2.5	0.3	9.4	9.7
2.8	T_8	2.8	2.7	2.5	3.9	10.3	6.6
3.6	T_1	3.6	3.3	3.7	10.7	1.5	13.6
3.0	T_2	3.0	3.2	3.7	6.9	25.2	17.1
3.4	T_3	3.4	3.7	3.7	8.2	9.4	1.1
3.6	T_4	3.6	3.6	3.7	2.4	4.2	1.7
3.7	T_5	3.7	3.5	3.7	5.7	1.2	7.2
3.3	T_6	3.3	3.4	3.7	2.9	10.7	7.6
3.4	T_7	3.4	3.6	3.7	4.9	7.9	2.9
3.5	T_8	3.5	3.6	3.7	1.8	4.5	2.7
6.0	T_1	6.0	5.6	5.7	6.1	4.5	1.7
5.7	T_2	5.7	6.0	5.7	4.6	0.7	5.1
5.9	T_3	5.9	6.5	5.7	10.1	3.3	12.2
6.1	T_4	6.1	5.8	5.7	5.0	6.7	1.8
6.2	T_5	6.2	5.8	5.7	6.9	8.5	1.7
5.5	T_6	5.5	5.8	5.7	5.4	3.2	2.2
6.1	T_7	6.1	5.7	5.7	7.0	7.2	0.2
6.1	T_8	6.1	6.3	5.7	3.3	6.7	9.7
8.1	T_1	8.1	8.5	7.7	4.2	5.3	9.2
8.9	T_2	8.9	8.8	7.7	0.4	13.1	12.8
8.3	T_3	8.3	8.5	7.7	2.7	6.8	9.2
8.5	T_4	8.5	8.0	7.7	6.6	9.9	3.6
8.1	T_5	8.1	8.3	7.7	2.8	4.7	7.3
9.3	T_6	9.3	9.5	7.7	2.6	17.3	19.3
8.6	T_7	8.6	8.6	7.7	0.3	10.5	10.3
9.2	T_8	9.2	9.2	7.7	0.6	16.4	15.9
1.9	T_1	1.9	1.9	1.7	0.0	9.4	9.4
1.7	T_2	1.7	1.7	1.7	1.7	0.9	2.5
1.8	T_3	1.8	1.9	1.7	5.5	8.0	12.8
1.6	T_4	1.6	1.6	1.7	1.0	3.3	4.4
1.7	T_5	1.7	1.6	1.7	7.6	0.1	8.1
1.8	T_6	1.8	1.9	1.7	3.2	7.5	10.4

Table S5. Summary of repeatability at fixed flow rates.

Pump Setting ($\mu L\ min^{-1}$)	n (Replicates)	Sensor Mean ($\mu L\ min^{-1}$)	Standard Deviation ($\mu L\ min^{-1}$)
1.8	8	1.9	0.1
1.3	8	1.5	0.1
2.5	8	2.7	0.1
3.7	8	3.4	0.2
5.7	8	6.0	0.2
7.7	6	8.6	0.5

Table S6. On-body validation of the sweat rate sensor via iontophoresis. The results compare sweat rate values obtained from the timer and sensor methods.

Subject	Sensor (µL / min)	Timer (µL / min)	RSD (%)
1	2.5	2.2	9.2
1	2.2	2.3	3.3
1	2.0	2.0	2.3
1	2.0	2.0	4.1
1	1.7	1.7	0
1	1.6	1.5	8.3
1	1.2	1.3	8.1
2	1.2	1.3	15.4
2	0.9	0.8	10.4
2	0.5	0.4	11.6

Table S7. On-body validation of the sweat rate sensor during cycling. The results compare sweat rate values obtained from the timer and sensor methods.

Subject	Sensor (µL / min)	Timer (µL / min)	RSD (%)
1	1.9	2.0	4.9
1	2.2	2.1	5.0
1	1.9	1.9	1.5
1	2.5	2.6	3.1
1	2.9	2.8	5.4
1	2.1	2.1	1.4
1	2.7	2.9	6.1
1	1.8	1.7	4.7
2	1.2	1.0	12.3
2	1.0	1.2	19.3
2	1.1	1.1	1.9
2	0.8	0.7	8.1

Table S8. The correlation between sweat rate sensor and cotton pad method in the on-body test with subject 1.

Sensor (µL / min)	Cotton patch (mL / min)	Correlation
1.2	54.0	0.788
1.1	52.0	
1.1	55.1	
0.6	46.5	

Table S9. The correlation between sweat rate sensor and cotton pad method in the on-body test with subject 2.

Sensor (μL / min)	Cotton patch (mL / min)	Correlation
2.0	49.0	0.927
2.2	51.1	
2.4	70.0	
2.2	50.5	

Table S10. Physiological parameters that were measured together with the sweat rate: the total sweat loss, the changes of forehead temperature (f_temp), ear temperature (e_temp), Borg scale, heart rate (HR), and urine density. Calculated values represent the difference between initial and final measurements for each subject.

**Subject**	**Total sweat lost** kg	**F temp** C°	**E temp** C°	**Borg**	**HR** BPM	**Urine Density (SG)**
1	0.62	0.6	1.0	9	40	0.001
2	0.66	0.1	1.5	6	44	0.0105

3. Python Code used in the simulation.

```
import numpy as np
import matplotlib.pyplot as plt

def p(*r):
    s = sum(1/x for x in r if x != 0)
    return 1/s if s != 0 else np.inf

# Parameters
sigma, Rcb = 0.17, 1000
w, h = 0.8e-3, 0.6e-3
A = w*h
L23, L35, L57 = 15.66e-3, 17.95e-3, 21.88e-3

Rexp = np.array([29.5e3,25.1e3,22.7e3,21.8e3,20.7e3,20.1e3,19.2e3])
steps = ['R01','R02','R03','R04','R05','R06','R07']

def sim(s, rcb):
    R01,R02,R03 = Rexp[:3]
    R12 = 1/(1/R02-1/R01)
    R23 = 1/(1/R03-1/R01-1/R12)
    Rs1=Rs2=0.5*(L35/(s*A))
    Rs3=Rs5=0.5*(L57/(s*A))
    Rs4=0.5*(L23/(s*A))
    R34 = p(R12/2, Rs1+rcb)+Rs2
    R56 = p(R23/2, Rs3+rcb)+Rs4
    return np.array([
        R01,
        p(R01,R12),
        p(R01,R12,R23),
        p(R01,R23,R34),
        p(R01,R12,R23,Rs1+Rs2),
        p(R01,R12,Rs1+Rs2,R56),
        p(R01,R12,R23,Rs1+Rs2,Rs3+Rs5)
    ])

Rsim = sim(sigma,Rcb)/1000
Rexp = Rexp/1000

plt.figure()
plt.plot(steps,Rexp,"o-",lw=2,label="Experimental")
plt.plot(steps,Rsim,"s--",lw=2,label=f"Simulation (Rcb={Rcb/1000} kΩ)")
plt.ylabel("Total Resistance (kΩ)")
plt.title(f"Carbon Black Simulation\nσ = {sigma} S/m")
plt.legend()
plt.grid(False)
plt.tight_layout()
plt.show()
```

4. References

(1) Islam, M. S.; Cha, S.; Hassan, M. F.; Cai, W.; Saniat, T. S.; Leach, C. R.; Khan, Y. Printed wearable sweat rate sensor for continuous in situ perspiration measurement. *Advanced Intelligent Systems* **2025**, *7* (3), 2400927.

(2) Liu, M.; Liu, S.; Zhang, T.; Zhou, D.; Li, L.; Gao, Q.; Liu, Y.; Ge, C.; Wang, Y.; Wang, M. Adaptively resettable microfluidic patch for sweat rate and electrolytes detection. *Biosensors and Bioelectronics* **2024**, *257*, 116299.

(3) Baker, L. B.; Model, J. B.; Barnes, K. A.; Anderson, M. L.; Lee, S. P.; Lee, K. A.; Brown, S. D.; Reimel, A. J.; Roberts, T. J.; Nuccio, R. P. Skin-interfaced microfluidic system with personalized sweating rate and sweat chloride analytics for sports science applications. *Science advances* **2020**, *6* (50), eabe3929.

(4) Choi, D.-H.; Gonzales, M.; Kitchen, G. B.; Phan, D.-T.; Searson, P. C. A capacitive sweat rate sensor for continuous and real-time monitoring of sweat loss. *ACS sensors* **2020**, *5* (12), 3821-3826.

(5) Aggarwal, A.; Dautta, M.; Ayala-Cardona, L. F.; Wudaru, A.; Javey, A. Wearable humidity sensor for continuous sweat rate monitoring. *Advanced Materials Technologies* **2023**, *8* (17), 2300385.

(6) Ria, A.; Piotto, M.; Muñoz-Berbel, X.; Bruschi, P.; Dei, M. Low-cost sweating-rate sensor for dehydration monitoring in sports. In *2023 IEEE SENSORS*, **2023**; IEEE: pp 1-4.

(7) Wei, L.; Lv, Z.; He, Y.; Cheng, L.; Qiu, Y.; Huang, X.; Ding, C.; Wu, H.; Liu, A. In-situ admittance sensing of sweat rate and chloride level in sweat using wearable skin-interfaced microfluidic patch. *Sensors and Actuators B: Chemical* **2023**, *379*, 133213.

(8) Honda, S.; Tanaka, R.; Matsumura, G.; Seimiya, N.; Takei, K. Wireless, flexible, ionic, perspiration-rate sensor system with long-time and high sweat volume functions toward early-stage, real-time detection of dehydration. *Advanced Functional Materials* **2023**, *33* (44), 2306516.
